# Recurrence-Free Survival as a Surrogate for Overall Survival Among Patients with Intrahepatic Cholangiocarcinoma Following Upfront Surgery: An International Multi-institutional Analysis

**DOI:** 10.1245/s10434-025-17156-5

**Published:** 2025-03-21

**Authors:** Jun Kawashima, Yutaka Endo, Selamawit Woldesenbet, Mujtaba Khalil, Miho Akabane, François Cauchy, Feng Shen, Shishir Maithel, Irinel Popescu, Minoru Kitago, Matthew J. Weiss, Guillaume Martel, Carlo Pulitano, Luca Aldrighetti, George Poultsides, Andrea Ruzzente, Todd W. Bauer, Ana Gleisner, Hugo Marques, Bas Groot Koerkamp, Itaru Endo, Timothy M. Pawlik

**Affiliations:** 1 Department of Surgery, The Ohio State University Wexner Medical Center and James Comprehensive Cancer Center, Columbus, OH USA; 2 Department of Gastroenterological Surgery, Yokohama City University School of Medicine, Yokohama, Japan; 3 Department of Transplant Surgery, University of Rochester Medical Center, Rochester, NY USA; 4 Department of Hepatobiliopancreatic Surgery, APHP, Beaujon Hospital, Clichy, France; 5 Department of Surgery, Eastern Hepatobiliary Surgery Hospital, Shanghai, China; 6 Department of Surgery, Emory University, Atlanta, GA USA; 7 Department of Surgery, Fundeni Clinical Institute, Bucharest, Romania; 8 Department of Surgery, Keio University, Tokyo, Japan; 9 Department of Surgery, Northwell Health, New Hyde Park, NY USA; 10 Department of Surgery, University of Ottawa, Ottawa, ON Canada; 11 Department of Surgery, Royal Prince Alfred Hospital, Camperdown, NSW Australia; 12 Department of Surgery, Ospedale San Raffaele, Milan, Italy; 13 Department of Surgery, Stanford University, Stanford, CA USA; 14 Department of Surgery, University of Verona, Verona, Italy; 15 Department of Surgery, University of Virginia, Charlottesville, VA USA; 16 Department of Surgery, University of Colorado Denver, Denver, CO USA; 17 Department of Surgery, Curry Cabral Hospital, Lisbon, Portugal; 18 Department of Surgery, Erasmus University Medical Centre, Rotterdam, The Netherlands

## Abstract

**Introduction:**

The role of recurrence-free survival (RFS) as a validated surrogate endpoint for overall survival (OS) among patients undergoing upfront surgery for intrahepatic cholangiocarcinoma (ICC) has not been defined. We sought to evaluate the correlation between RFS and OS after surgical resection for ICC. We hypothesized that RFS was a reliable surrogate endpoint for OS among patients with ICC.

**Methods:**

Patients who underwent upfront curative-intent surgery for ICC between 2000 and 2023 were identified from an international, multi-institutional database. The correlation between RFS and OS was assessed using rank correlation. Landmark analysis evaluated concordance between survival at 5 years and recurrence status at 6, 12, 24, 36, 48, and 54 months postoperatively.

**Results:**

Among 1541 patients who underwent curative-intent hepatic resection, the median RFS and OS were 22.6 months and 41.5 months, respectively. A moderately strong correlation between RFS and OS was identified (*ρ* = 0.79, 95% CI 0.76 to 0.82). In the landmark analysis, the concordance between 5-year OS after surgery and recurrence status at different time points (6, 12, 24, 36, 48, and 54 months) was 60.7%, 72.0%, 81.4%, 83.1%, 83.0%, and 82.5%, respectively. Restricted cubic spline analysis indicated that the prediction of OS based on RFS increased with time and plateaued 3 years after surgery.

**Conclusions:**

Among patients undergoing curative-intent resection of ICC, there was a moderately strong correlation between RFS and OS. Three-year RFS may be a reliable surrogate endpoint to predict 5-year OS and should be considered in future trial design.

**Supplementary Information:**

The online version contains supplementary material available at 10.1245/s10434-025-17156-5.

Intrahepatic cholangiocarcinoma (ICC) is the second most common type of primary liver cancer, accounting for roughly 10-20% of all hepatic tumors.^1^ Over the past few decades, the global incidence of ICC has been increasing, representing a growing public health challenge.^2,3^ Surgical resection remains the only potentially curative treatment option for ICC; however, the prognosis for resectable ICC is still poor with 5-year overall survival (OS) ranging from 20-35%.^4^ Additionally, despite curative-intent surgery, the incidence of recurrence is alarmingly high with around 80% of patients experiencing recurrence within two years post-resection and 25% experiencing a recurrence within just six months.^5,6^ These statistics highlight an urgent need for improved treatment strategies, including multidisciplinary approaches that involve perioperative systemic chemotherapy, targeted therapies, and immunotherapies.^4^

Several randomized controlled trials (RCTs) have been conducted among patients with biliary tract cancers (BTCs) including ICC to investigate the potential benefits of perioperative adjuvant therapies.^7,8^ While OS is traditionally regarded as the gold standard primary endpoint in cancer therapy trials, relying on OS has notable limitations.^9^ Of note, its requirement for larger patient cohorts and longer follow-up periods can delay the development and clinical application of new therapies.^10^ Consequently, there is a growing need to identify alternative surrogate endpoints that are both statistically robust and clinically relevant.^11^ Recurrence-free survival (RFS) has emerged as a promising alternative endpoint, offering multiple advantages, such as shorter follow-up durations and faster trial completion, which may lead to earlier access to new treatments, enhanced trial efficiency, and cost savings.^9^ For instance, in colorectal cancer, disease-free survival (DFS) has become the primary endpoint in several RCTs of adjuvant therapy.^12^ Specifically, among patients with colorectal cancer, a 3-year DFS has been established as a reliable substitute for traditional 5-year OS.^12^ Moreover, DFS has been validated as a surrogate endpoint for OS in other cancers, including gastric, renal, and lung cancers, suggesting its potential to streamline clinical trials and conserve resources.^13–15^ However, despite the success of these surrogate endpoints in various cancers, research on the application of RFS as a validated endpoint among patients with ICC remains limited.^9^

To date, ICC has often been included in RCTs as part of a heterogeneous group of patients with other bile duct and gallbladder cancers due to the low incidence of ICC.^4^ While this approach has provided some insights, the heterogeneity of the patient population can obscure the effects of targeted treatments specific to ICC. To facilitate the development and application of promising therapies tailored to ICC, it is crucial to conduct efficient clinical trials focusing exclusively on ICC patients.^4^ Therefore, the current study sought to evaluate the correlation between RFS and OS after surgical resection for ICC using data from a large, international, multi-institutional database. We hypothesized that RFS was a reliable surrogate endpoint for OS among patients with ICC. If true, use of RFS in future clinical trials may help accelerate the testing and delivery of new therapies for patients with ICC.

## Methods

### Data Source and Patient Selection

Patients who underwent curative-intent liver resection for ICC between 2000 and 2023 were identified from the International Intrahepatic Cholangiocarcinoma Study Group database (Supplementary Table 1).^16^ The database consisted of retrospectively collected data from participating institutions across the globe. Each participating institution was responsible for collecting and auditing its own data. At The Ohio State University Wexner Medical Center, data from each participating institution were merged, re-audited, and securely stored in a dedicated institutional database for research purposes. The most recent update, which included data up to December 2023, was used for the current study. Patients were excluded based on (1) receipt of preoperative systemic chemotherapy, (2) macroscopically positive surgical margins (R2 resection), (3) extrahepatic metastasis, and (4) lack of follow-up data. The Institutional Review Boards of each participating institution approved the study.

### Variables and Outcomes

Patient demographic and clinicopathologic variables included age, sex, American Society of Anesthesiologists Physical Status (ASA PS) classification, geographic region (i.e., USA/Canada, Europe, Australia, Asia), year of surgery (i.e., 2000-2010, 2011-2023), pathological tumor size and number, T-category based on AJCC 8th edition,^17^ nodal diseases (i.e. N0: negative, Nx: not examined, N1: positive), microvascular invasion (MVI), surgical margin (i.e., R0, R1), and adjuvant chemotherapy. In this study, multifocal disease included both satellite lesions, defined as tumors within the same Couinaud liver segment, and intrahepatic metastases, defined as tumors located in different Couinaud liver segments.^17^ Due to the retrospective nature of this study, distinguishing between intrahepatic metastases and multicentric tumors was not feasible.

OS was defined as the time interval between the date of liver resection for ICC and the date of death or last follow-up. RFS was defined as the time elapsed between the date of liver resection and recurrence, confirmed either on biopsy or based on evidence of a suspicious lesion on follow-up imaging. Survival after recurrence (SAR) was calculated only in cases that experienced recurrence and was defined as the date of recurrence to the data of death from any cause. Following curative-intent hepatectomy, patients were monitored for recurrence based on serum tumor markers and imaging including CT and/or MRI. Patients were followed once every 3 months during the first 3 years, once every 6 months during the 4^th^ and 5^th^ years, and then annually thereafter.^16^While the overall follow-up strategy was generally aligned with the described schedule, follow-up intervals varied among centers due to differences in institutional protocols and regional practices. The last follow-up date was December 26, 2023. The treatment of tumor recurrence was decided based on consensus among the multidisciplinary team at each institution. The primary outcome of interest was the correlation between RFS and OS. Furthermore, a landmark analysis was performed to determine the number of years of RFS that was an appropriate alternative to 5-year OS.

### Statistical Analysis

The median follow-up period (95% CI) was determined using the reverse Kaplan-Meier method, while OS and RFS were estimated using the standard Kaplan-Meier method.^9^ To assess the correlation between OS and RFS, Spearman correlation analysis was conducted. The correlation coefficient (ρ) was classified following the guidelines proposed by Prasad et al.: low correlation (0.7 or below), moderately strong correlation (greater than 0.70 but less than 0.85), and strong correlation (0.85 or above).^18,19^ For the landmark analysis, concordance of 5-year survival among patients without recurrence and 5-year mortality among individuals with recurrence was evaluated at various time points (6, 12, 24, 36, 48, and 54 months).^9^ Restricted cubic splines (RCS) with four knots were utilized to examine changes in concordance. In addition, to interrogate the potential effect of adjuvant chemotherapy, the correlation between RFS and OS was examined according to adjuvant chemotherapy. Furthermore, a subgroup analysis stratified by geographic regions into Western countries (USA/Canada, Europe, Australia) and Eastern countries (Asia) was conducted; patient characteristics were compared between these two groups. Subsequently, the correlation between RFS and OS was independently evaluated within the Western and Eastern cohorts.

Descriptive statistics were presented as median values with interquartile ranges (IQR) for continuous variables and as frequencies with percentages (%) for categorical variables. Survival was compared with log-rank test. Statistical significance was set at α = 0.05. All analyses were performed using R version 4.2.2 (R Project for Statistical Computing, Vienna, Austria).

## Results

### Baseline Cohort Characteristics

Among 1,591 patients who met the inclusion criteria, 875 (55.0%) were male with a median age of 62 years (IQR, 54 to 70); approximately one-half of patients (n = 818, 51.4%) were classified as ASA-PS 1 or 2. Most patients (n = 1,274, 80.1%) had a solitary tumor with a median size of 5.8 cm (IQR, 3.9 to 8.1). On final pathology, 596 (37.5%) patients had T2 disease and 347 (21.8%) had lymph node metastasis (N1). Microvascular invasion (MVI) was present in 453 (28.5%) patients; 280 (17.6%) patients had an R1 margin status. In the post-operative setting, 436 (27.4%) received adjuvant chemotherapy (Table 1).

**Table 1:** Clinicopathological characteristics of the analytic cohort

Characteristics	All patients
	n = 1591
*Age, y, median (IQR)*	62 [54, 70]
*Sex, men*	875 (55.0)
*ASA- PS classification*	
1 or 2	818 (51.4)
> 2	638 (40.1)
Missing	135 (8.5)
*Geographic region*	
USA/Canada	460 (28.9)
Europe	573 (36.0)
Australia	95 (6.0)
Asia	463 (29.1)
*Year of surgery*	
2000-2010	616 (38.7)
2011-2023	975 (61.3)
*Number of tumors*	
Solitary lesions	1274 (80.1)
Multiple lesions	205 (12.9)
Missing	112 (7.0)
*Size of largest tumor, cm, median (IQR)*	5.8 [3.9, 8.1]
*Pathological T category*	
T1	499 (31.4)
T2	596 (37.5)
T3	372 (23.4)
T4	58 (3.6)
Missing	66 (4.1)
*Pathological N category*	
N0	503 (31.6)
N1	347 (21.8)
Nx	678 (42.6)
Missing	63 (4.0)
*Microvascular invasion*	
Yes	453 (28.5)
No	1030 (64.7)
Missing	108 (6.8)
*Margin, positive*	
R0	1311 (82.4)
R1	280 (17.6)
*Adjuvant chemotherapy*	208 (14.7)
Yes	436 (27.4)
No	1155 (72.6)

Values are n (%) unless otherwise indicated

ASA PS, American society of anesthesiologists physical status

### Survival and the Correlation Between Recurrence-Free Survival and Overall Survival

**Fig. 1:**
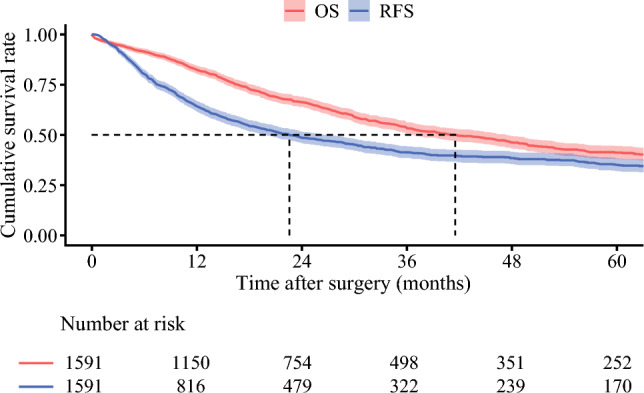
Kaplan-Meier estimates of recurrence-free survival (RFS) and overall survival (OS) for the entire cohort

**Fig. 2:**
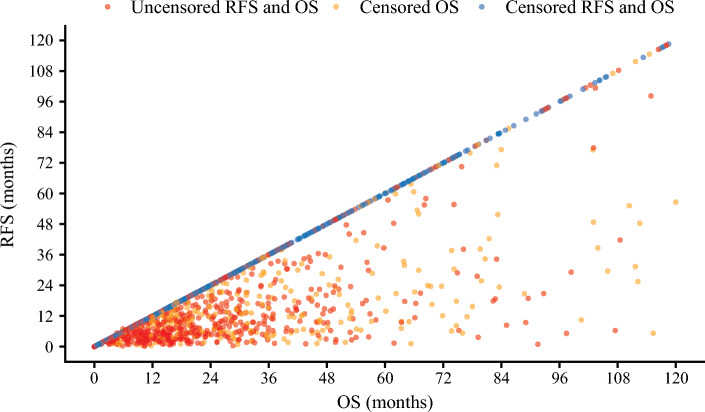
Scatter plot depicting the relationship between recurrence-free survival (RFS) and overall survival (OS) times in the entire cohort

The median duration of follow-up was 45.8 months (95%CI 42.0 to 49.3). A total of 816 (51.3%) patients experienced a recurrence and 765 (48.1%) patients had died at the time of last follow-up. Median RFS was 22.6 months (95%CI 19.9 to 26.4) with a 3-year RFS of 41.2% (95%CI 38.5 to 44.2); median OS was 41.5 months (95%CI 36.9 to 47.2) with a 5-year OS of 41.5% (95%CI 36.9 to 47.2) (Fig. 1). Median SAR was 16.4 months (95%CI 14.4 to 18.0) among patients with a recurrence (Supplementary Fig. 1). In the primary analysis of patients in the entire cohort, there was a moderately strong correlation between RFS and OS (ρ = 0.79, 95%CI 0.76 to 0.82) (Fig. 2).

### The Landmark Analysis for Predicting Death or Survival at 5 Years

**Fig. 3:**
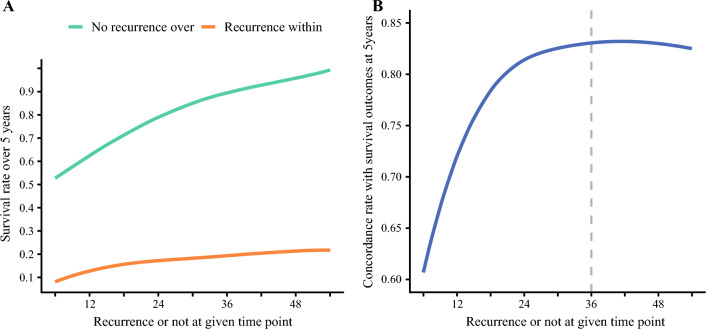
Landmark analysis evaluating the prediction of survival at 5 years post-surgery based on recurrence status at specific time points. **A** 5-year overall survival rate among patients with or without recurrence at each time point. **B** Concordance rate for 5-year survival in patients without recurrence and 5-year mortality in those who experienced recurrence at each time point

Survival at 5 years after resection was predicted using a landmark analysis stratified by the presence or absence of recurrence at each time point. Among patients with recurrence within 6, 12, 24, 36, 48, and 54 months following the index hepatic resection, predicted survival at 5 years was 8.0% (95%CI 4.5 to 14.2), 13.2% (95%CI 9.7 to 18.0), 16.6% (95%CI 13.3 to 20.9), 19.9% (95%CI 16.4 to 24.0), 20.9% (95%CI 17.5 to 25.0), and 21.9% (95%CI 18.5 to 26.0), respectively. In contrast, among patients who did not experience a recurrence at each time point, the estimated survival of individuals who remained alive at 5 years after resection was 52.6% (95%CI 49.0 to 56.4), 62.8% (95%CI 58.8 to 67.1), 78.5% (95%CI 74.4 to 82.9), 89.9% (95%CI 86.3 to 93.6), 95.5% (95%CI 92.9 to 98.3), and 99.5% (95%CI 98.5 to 100.0), respectively. The RCS plot demonstrated that prognosis was particularly poor among patients with recurrence within 24 months. However, prognosis did improve rapidly among patients who had longer recurrence-free periods even over the initial 6 to 24 months after resection (Fig. 3A). The concordance of 5-year survival among patients without recurrence and 5-year mortality among individuals with recurrence at each time point was 60.7% (95%CI 56.5 to 64.4), 72.0% (95%CI 67.7 to 76.0), 81.4% (95%CI 77.2 to 85.1), 83.1% (95%CI 79.1 to 86.6), 83.0% (95%CI 79.0 to 86.3), and 82.5% (95%CI 79.0 to 85.1), respectively. The RCS plot indicated that the prediction of OS based on RFS increased with time and plateaued 3 years after surgery (Fig. 3B). Of note, patients with recurrence within 3 years had a worse prognosis after recurrence compared with individuals who had a recurrence after 3 years (median SAR, 16.0 months [95%CI 14.1 to 17.6] vs 66.7 months [95%CI 21.1 to not reached], p < 0.001) (Supplementary Fig. 2).

### Additional Analysis Among Patients Who Received Adjuvant Chemotherapy

**Fig. 4:**
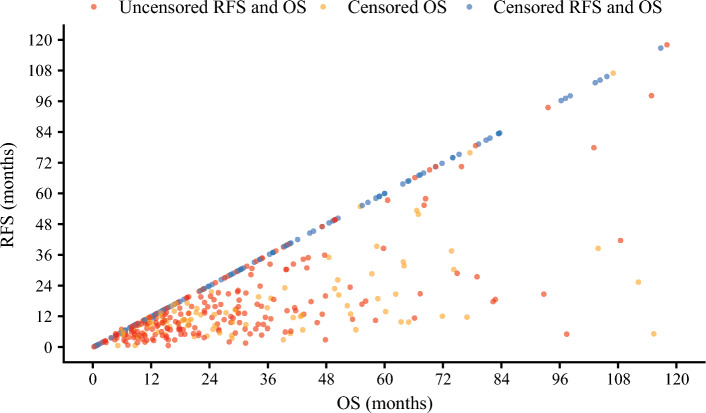
Scatter plot illustrating the relationship between recurrence-free survival (RFS) and overall survival (OS) times in patients who received adjuvant chemotherapy

To investigate the potential effect of adjuvant chemotherapy, a sensitive analysis was performed among patients who received adjuvant chemotherapy. Among 436 patients treated with postoperative adjuvant chemotherapy, 279 (64.0%) patients recurred and 223 (51.1%) patients had died at last follow-up. There was a moderately strong correlation between RFS and OS (ρ = 0.78, 95%CI 0.72 to 0.82) (Fig. 4).

### Additional Analysis Stratified by Geographic Region.

In the entire cohort, 1,128 (70.9%) patients were from Western countries, while 463 (29.1%) patients were from Eastern countries. The differences in clinicopathological characteristics between the two groups are summarized in Supplementary Table 2. Patients in Western countries were more likely to have adverse tumor features, such as larger tumor size, multifocality, nodal disease, MVI, and positive surgical margins. When the correlation between RFS and OS was analyzed by region, a strong correlation was observed among patients from Western countries (ρ = 0.85, 95% CI 0.82 to 0.87) (Supplementary Fig. 3); patients from Eastern countries demonstrated a lower correlation (ρ = 0.65, 95% CI 0.57 to 0.71) (Supplementary Fig. 4).

## Discussion

OS is widely regarded as the gold standard endpoint in oncological clinical trials.^9,11^ Relying on OS can present several challenges, however, including the need for larger sample sizes and a prolonged follow-up period, which can impede the pace of therapeutic drug development.^10,20^ In contrast, alternative endpoints such as RFS, which require less time to assess, can accelerate trial completion, enable earlier access to new treatments, as well as improve efficiency and reduce costs of clinical trials.^9^ RFS has previously been validated as a surrogate endpoint for OS among patients with colorectal, gastric, renal, and lung cancer – yet not ICC.^12–15^ ICC is a particularly aggressive cancer that requires a multidisciplinary treatment approach that incorporates resection and often perioperative systemic therapy.^9,21–23^ Despite several adjuvant trials that are underway, reliable surrogate endpoints for OS following surgical resection are still lacking in BTC, including ICC.^5–9^ As delays in clinical trials can hinder the timely adoption of new therapies, the validation of alternative endpoints is essential.^20^ Therefore, the current study was important because we identified a moderately strong correlation (ρ = 0.79, 95% CI 0.76 to 0.82) between RFS and OS among ICC patients who underwent curative-intent surgery using data from a large, international, multi-institutional database. Of particular note, the landmark analysis demonstrated that the predictive value of recurrence status for 5-year OS steadily increased and plateaued at 3 years, suggesting that 3-year RFS may serve as a reliable surrogate endpoint for 5-year OS among patients with ICC. These findings can hopefully inform the design of future clinical trials aimed at identifying new therapies for ICC patients following hepatic resection.

The use of RFS as a surrogate endpoint for OS was initially validated based on analyses of the Adjuvant Colon Cancer Endpoints (ACCENT) database, which pooled data from RCTs of fluorouracil-based adjuvant therapy for non-metastatic colorectal cancer.^12^ These analyses established that 3-year RFS could serve as a surrogate for 5-year OS.^12^ In the field of BTC, a previous report demonstrated a Spearman rank correlation between RFS and OS of 0.75 at the patient-level analysis and 0.87 at the trial-level analysis, suggesting that RFS can be a reliable surrogate for OS in this patient population.^9^ Similarly, a meta-analysis by Moriwaki et al. noted a strong correlation between progression-free survival (PFS) and OS, further supporting PFS as a valid surrogate marker for OS among individuals with advanced BTC.^24^ Consistent with these findings, the current study reported a Spearman rank correlation coefficient of 0.79 (95%CI 0.76 to 0.82) in patient-level analysis, indicating a moderately strong correlation between RFS and OS among patients who underwent upfront curative-intent resection of ICC. SAR might play a key role in the correlation between RFS and OS.^25^ The strength of this correlation is likely influenced by the effectiveness of treatments available after recurrence.^9,25^ For instance, Imamura et al. noted that diseases with shorter median SAR, such as pancreatic ductal adenocarcinoma and BTC, tend to have a stronger correlation between RFS and OS.^9^ In contrast, cancers like hepatocellular carcinoma and colorectal liver metastases, which have more established post-recurrence treatment options and consequently longer SAR, tend to have a weaker correlation between RFS and OS.^9,25,26^ Given the aggressive nature of ICC, SAR is particularly poor.^5,27^ In the current study, the median SAR for ICC was only 16.4 months, consistent with previous studies that reported a range of 10 to 18 months.^5,27^ This relatively short SAR suggests that patients with ICC often do not survive long enough after recurrence for post-recurrence treatments to alter their prognosis. As a result, RFS may be a reliable surrogate endpoint for OS in the setting of ICC, since recurrence itself is so closely linked with poor OS outcomes.

Approximately 80% of ICC patients who recur experience the recurrence within the first 2 years after surgery, with these patients having a much worse prognoses versus individuals who recur after 2 years.^5^ In fact, ICC patients with early recurrence (i.e. recurrence within 2 years after surgery) had 1.89 times worse SAR compared with individuals with late recurrence.^27^ In the current study, the vast majority of recurrences (n = 754, 91.2%) occurred within the first 3 years after hepatic resection and these individuals had a 5-year survival of only 20%. In contrast, patients who survived without recurrence over the first 3 years had a nearly 90% chance of 5-year survival. Additionally, patients with recurrence within 3 years had worse SAR compared with individuals who recurred after 3 years (Supplementary Fig. 2). These findings highlight that the prognosis of ICC patients is heavily influenced by both the presence and timing of recurrence. The landmark analysis further validated this point as the impact of recurrence status to predict 5-year OS steadily increased and plateaued at 3 years. In turn, these data suggest that 3-year RFS can serve as a reliable surrogate endpoint for 5-year OS among patients undergoing resection of ICC. This observation was consistent with another report that examined BTCs in which recurrence at 3 years post-surgery strongly predicted 5-year OS.^9^ By establishing 3-year RFS as a surrogate endpoint, clinical trials can benefit from earlier completion and cost savings.^9^ Moreover, reducing the endpoint from 5 years to 3 years may directly benefit patients by allowing faster implementation of novel treatment strategies, thereby improving clinical outcomes sooner.

Recent evidence has increasingly supported the role of adjuvant treatment to improve postoperative survival for patients with BTCs.^7,8,28^ For example, the BILCAP study demonstrated a survival benefit with adjuvant capecitabine following curative resection of BTCs.^28^ Adjuvant capecitabine was associated with an adjusted OS hazard ratio of 0.74 (95%CI 0.59 to 0.94) and an RFS hazard ratio of 0.77 (95%CI 0.61 to 0.97) in the per-protocol analysis.^28^ Similarly, the ASCOT trial identified a survival benefit with adjuvant chemotherapy using S-1 in BTC patients after surgery.^8^ In most adjuvant trials for BTCs, OS was used as the primary endpoint, and few clinical trials to date have utilized RFS as a surrogate for OS in this context.^7,8,28–30^ Only one meta-analysis has directly investigated RFS as a surrogate endpoint in adjuvant therapy trials for BTCs, which reported a strong correlation between RFS and OS (Spearman rank coefficient of 0.87).^9^ In line with these data, we similarly noted a moderately strong correlation between RFS and OS among patients with ICC who received adjuvant chemotherapy. This finding is important, as it indicates that RFS remains a robust predictor of long-term survival outcomes, even in the context of adjuvant therapy in which therapeutic interventions may delay or prevent recurrence. Beyond adjuvant chemotherapy, there is growing interest in neoadjuvant chemotherapy, targeted therapy, and immunotherapy.^31–34^ Further refinements in the surgical management of ICC combined with advances in locoregional treatments and novel systemic therapies are expected to enhance patient outcomes.^4,35^ Collectively, the data from previous work and the current study, strong suggest that RFS can be used as reliable surrogate endpoint for OS in perioperative ICC trials involving surgical patients.

Several limitations should be acknowledged when interpreting the results of the current study. Although a strength, the use of a large, international, multi-institutional database also introduced heterogeneity in patient selection, treatment approaches, and follow-up protocols across different centers. Specifically, variations in the administration and types of adjuvant chemotherapy, as well as differences in surgical techniques, may have influenced the outcomes. Furthermore, treatment after recurrence—which can significantly impact OS—varied among institutions, potentially affecting the observed correlation between RFS and OS. Notably, the correlation between RFS and OS differed somewhat between Western and Eastern cohorts, with a lower correlation observed in Eastern countries. This discrepancy may be due to differences in tumor biology, such as a higher proportion of favorable tumors in Eastern countries, smaller sample size, and variations in post-recurrence treatment strategies. The rarity of ICC required data collection over an extended period, which could have introduced temporal biases related to evolving treatment strategies and surgical practices. Additionally, while this study focused on patient-level correlation, a trial-level analysis is necessary to fully validate 3-year RFS as a surrogate endpoint for OS in clinical trials. Future research should address these limitations by including prospective data and standardizing postoperative treatment protocols to confirm the robustness of RFS as a surrogate endpoint in different clinical settings.

In summary, there was a moderately strong correlation between RFS and OS among ICC patients undergoing curative-intent hepatic resection, irrespective of adjuvant chemotherapy administration. Importantly, the landmark analysis identified that 3-year RFS may serve as a reliable surrogate endpoint for 5-year OS offering a potential means to accelerate clinical trial completion and facilitate earlier adoption of new therapeutic strategies. These data are particularly significant for ICC, an aggressive cancer with limited treatment options. The use of surrogate endpoints such as RFS may provide a pathway to optimize clinical trial design and expedite the development of multidisciplinary treatments for ICC.

## Supplementary Information

Below is the link to the electronic supplementary material.

Supplementary file1 (DOCX 40 KB)

Supplementary file2 (TIF 262 KB)

Supplementary file3 (TIF 312 KB)

Supplementary file4 (TIF 397 KB)

Supplementary file5 (TIF 347 KB)

Supplementary file6 (DOCX 15 KB)
