# Supplementary material for: Recurrence-Free Survival as a Surrogate for Overall Survival Among Patients with Intrahepatic Cholangiocarcinoma Following Upfront Surgery: An International Multi-institutional Analysis
Source: Ann Surg Oncol. 2025 Mar 21;32(7):4967–75. doi: 10.1245/s10434-025-17156-5 (PMC12130119; doi:10.1245/s10434-025-17156-5)
Supplement: Supplementary file 1 — Supplementary file1 (DOCX 40 KB) [file 10434_2025_17156_MOESM1_ESM.docx]

**Supplementary Table 1**. Patient distribution across participating institutions

| Institutions |  |
| --- | --- |
| *USA/Canada* |  |
| Northwell Health, USA | 20 (1.3) |
| Cleveland Clinic Foundation, USA | 88 (5.5) |
| Emory University, USA | 122 (7.7) |
| Johns Hopkins University, USA | 79 (5.0) |
| Stanford University, USA | 56 (3.5) |
| University of Colorado Denver, USA | 21 (1.3) |
| University of Virginia, USA | 22 (1.4) |
| University of Ottawa, Canada | 57 (3.6) |
| *Europe* |  |
| Beaujon Hospital, France | 62 (3.9) |
| Curry Cabral Hospital, Portugal | 91 (5.7) |
| Erasmus University Medical Centre, The Netherlands | 109 (6.9) |
| Fundeni Clinical Institute, Romania | 177 (11.1) |
| University of Verona, Italy | 134 (8.4) |
| *Australia* |  |
| The University of Sydney, Australia | 48 (3.0) |
| Royal Prince Alfred Hospital, Australia | 47 (3.0) |
| *Asia* |  |
| Eastern Hepatobiliary Surgery Hospital, China | 371 (23.3) |
| Keio University, Japan | 25 (1.6) |
| Yokohama City University, Japan | 67 (4.2) |

**Supplementary Table 2**. Comparison of patient characteristics between Western and Eastern cohorts

| Characteristics | Western countries | Eastern countries | p-value |
| --- | --- | --- | --- |
|  | n = 1128 (70.9%) | n = 463 (29.1%) |  |
| Age, y, median (IQR) | 64 [56, 71] | 57 [48, 65] | <0.001 |
| Sex, men | 560 (49.6) | 315 (68.0) | <0.001 |
| ASA- PS classification |  |  | <0.001 |
| 1 or 2 | 362 (32.1) | 456 (98.5) |  |
| > 2 | 632 (56.0) | 6 (1.3) |  |
| Missing | 134 (11.9) | 1 (0.2) |  |
| Year of surgery |  |  | 0.754 |
| 2000-2010 | 440 (39.0) | 176 (38.0) |  |
| 2011-2023 | 688 (61.0) | 287 (62.0) |  |
| Number of tumors |  |  | <0.001 |
| Solitary lesions | 839 (74.4) | 435 (94.0) |  |
| Multiple lesions | 177 (15.7) | 28 (6.0) |  |
| Missing | 112 (9.9) | 0 (0.0) |  |
| Size of largest tumor, cm, median (IQR) | 6.0 [4.0, 9.0] | 5.1 [3.5, 7.1] | <0.001 |
| Pathological T category |  |  | <0.001 |
| T1 | 273 (24.2) | 226 (48.8) |  |
| T2 | 478 (42.4) | 118 (25.5) |  |
| T3 | 264 (23.4) | 108 (23.3) |  |
| T4 | 47 (4.2) | 11 (2.4) |  |
| Missing | 66 (5.9) | 0 (0.0) |  |
| Pathological N category |  |  | <0.001 |
| N0 | 405 (35.9) | 98 (21.2) |  |
| N1 | 278 (24.6) | 69 (14.9) |  |
| Nx | 382 (33.9) | 296 (63.9) |  |
| Missing | 63 (5.6) | 0 (0.0) |  |
| Microvascular invasion |  |  | <0.001 |
| Yes | 357 (31.6) | 96 (20.7) |  |
| No | 676 (59.9) | 354 (76.5) |  |
| Missing | 95 (8.4) | 13 (2.8) |  |
| Margin, positive |  |  | <0.001 |
| R0 | 870 (77.1) | 441 (95.2) |  |
| R1 | 258 (22.9) | 22 (4.8) |  |
| Adjuvant chemotherapy |  |  | <0.001 |
| Yes | 387 (34.3) | 49 (10.6) |  |
| No | 741 (65.7) | 414 (89.4) |  |

Values are n (%) unless otherwise indicated.

**ASA PS**, American Society of Anesthesiologists Physical Status
