# Supplementary figures and images for: Recurrence-Free Survival as a Surrogate for Overall Survival Among Patients with Intrahepatic Cholangiocarcinoma Following Upfront Surgery: An International Multi-institutional Analysis
Source: Ann Surg Oncol. 2025 Mar 21;32(7):4967–75. doi: 10.1245/s10434-025-17156-5 (PMC12130119; doi:10.1245/s10434-025-17156-5)

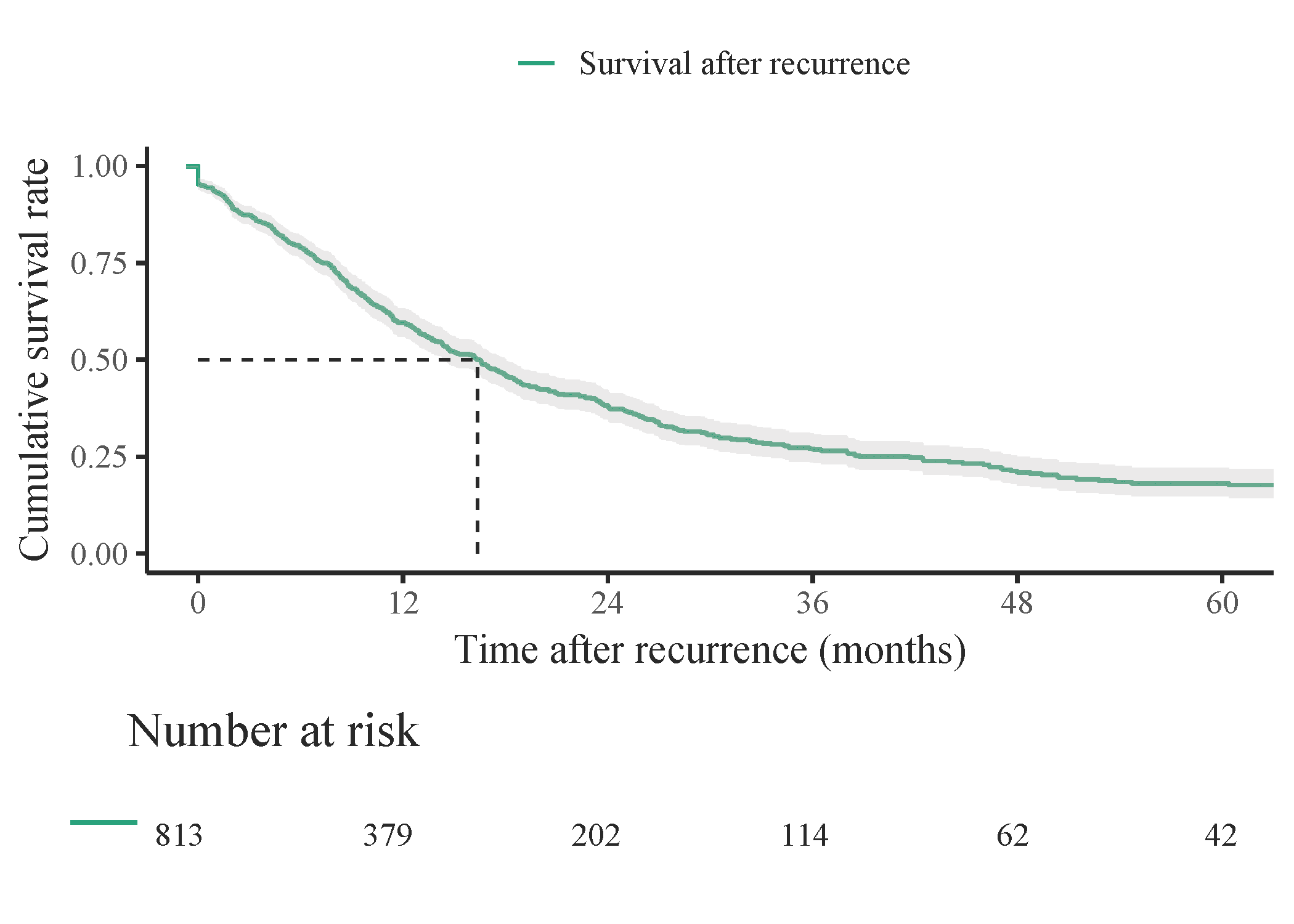

Supplement: Supplementary file 2 — Supplementary file2 (TIF 262 KB) [file 10434_2025_17156_MOESM2_ESM.tif]

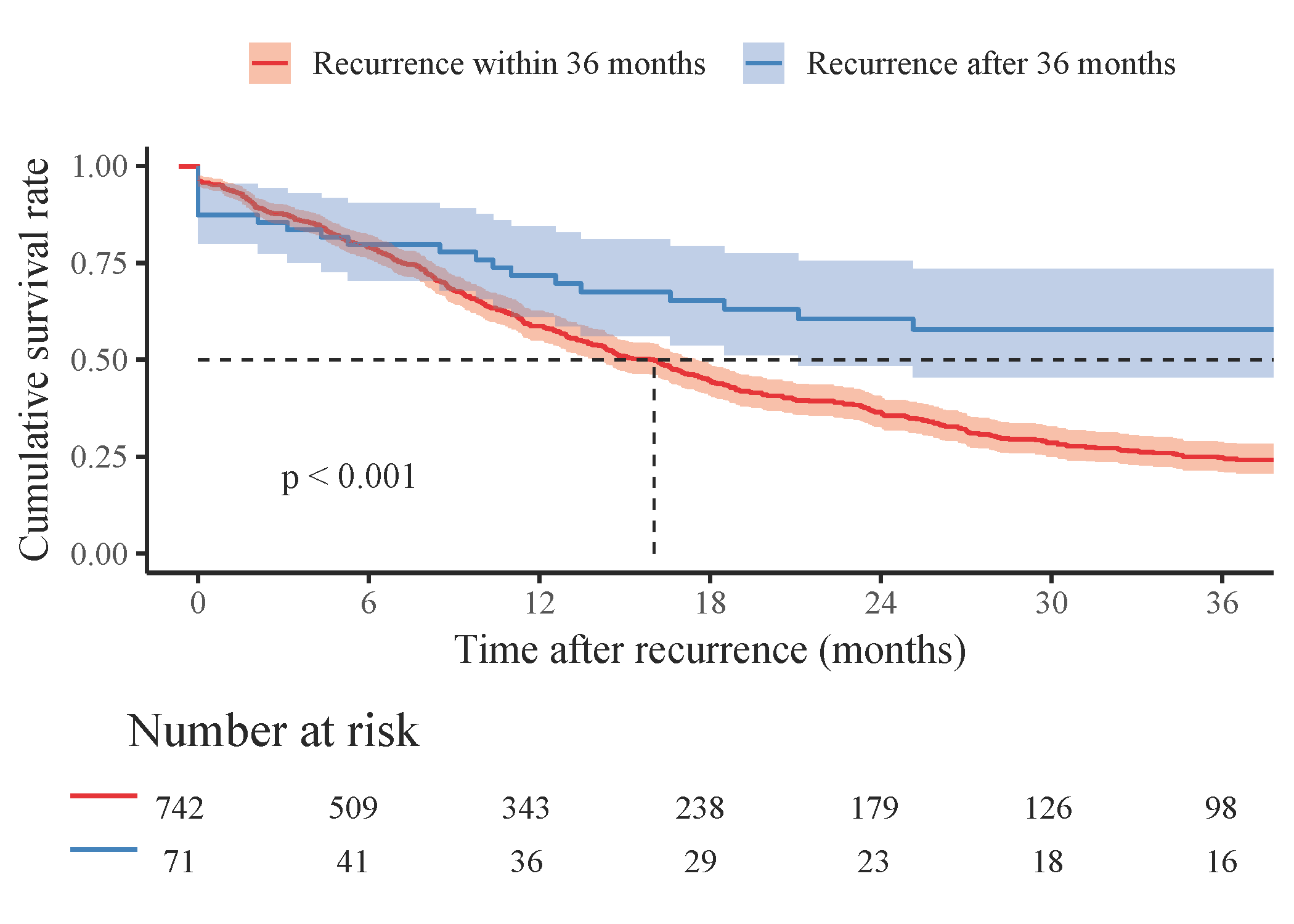

Supplement: Supplementary file 3 — Supplementary file3 (TIF 312 KB) [file 10434_2025_17156_MOESM3_ESM.tif]

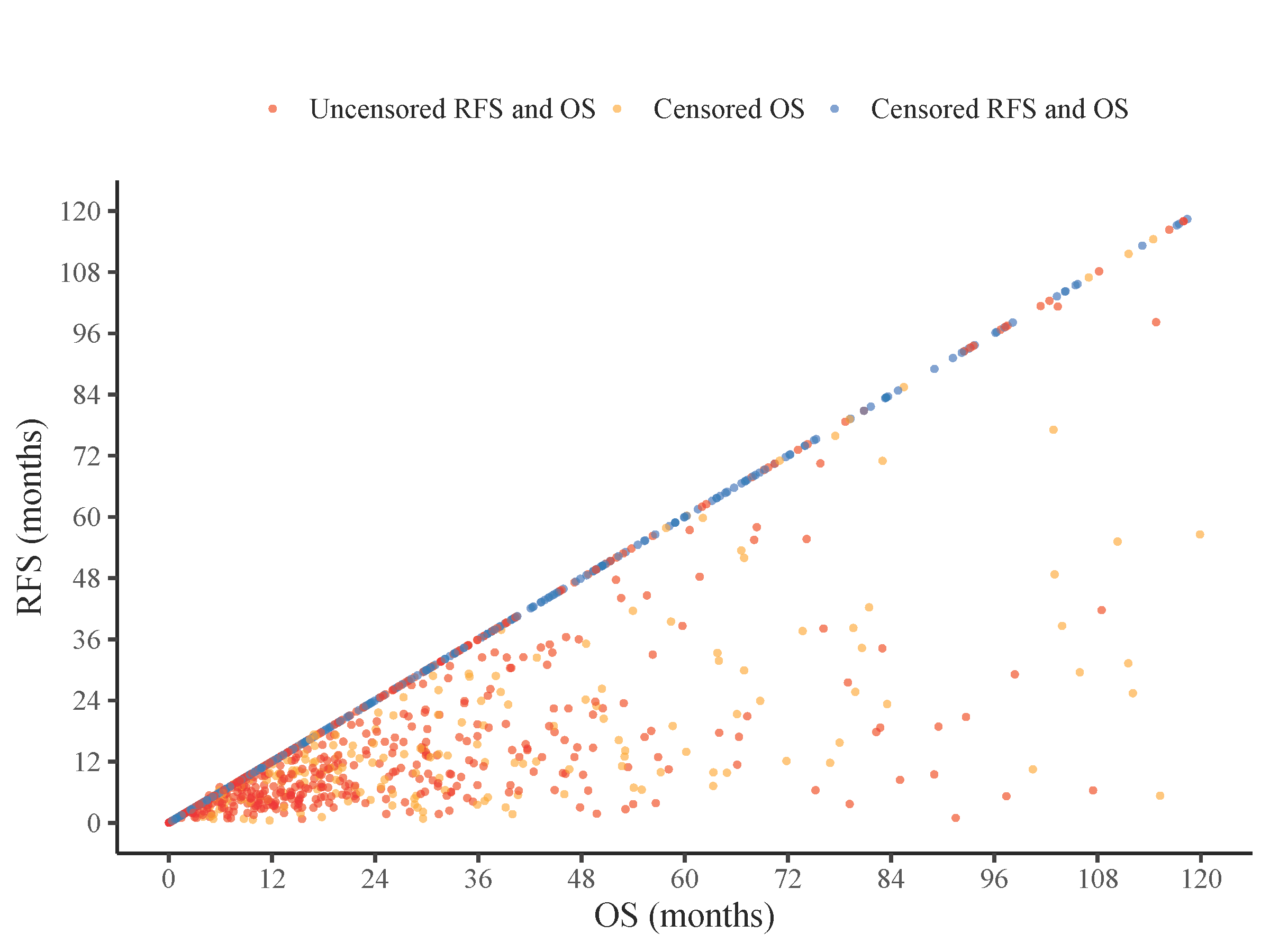

Supplement: Supplementary file 4 — Supplementary file4 (TIF 397 KB) [file 10434_2025_17156_MOESM4_ESM.tif]

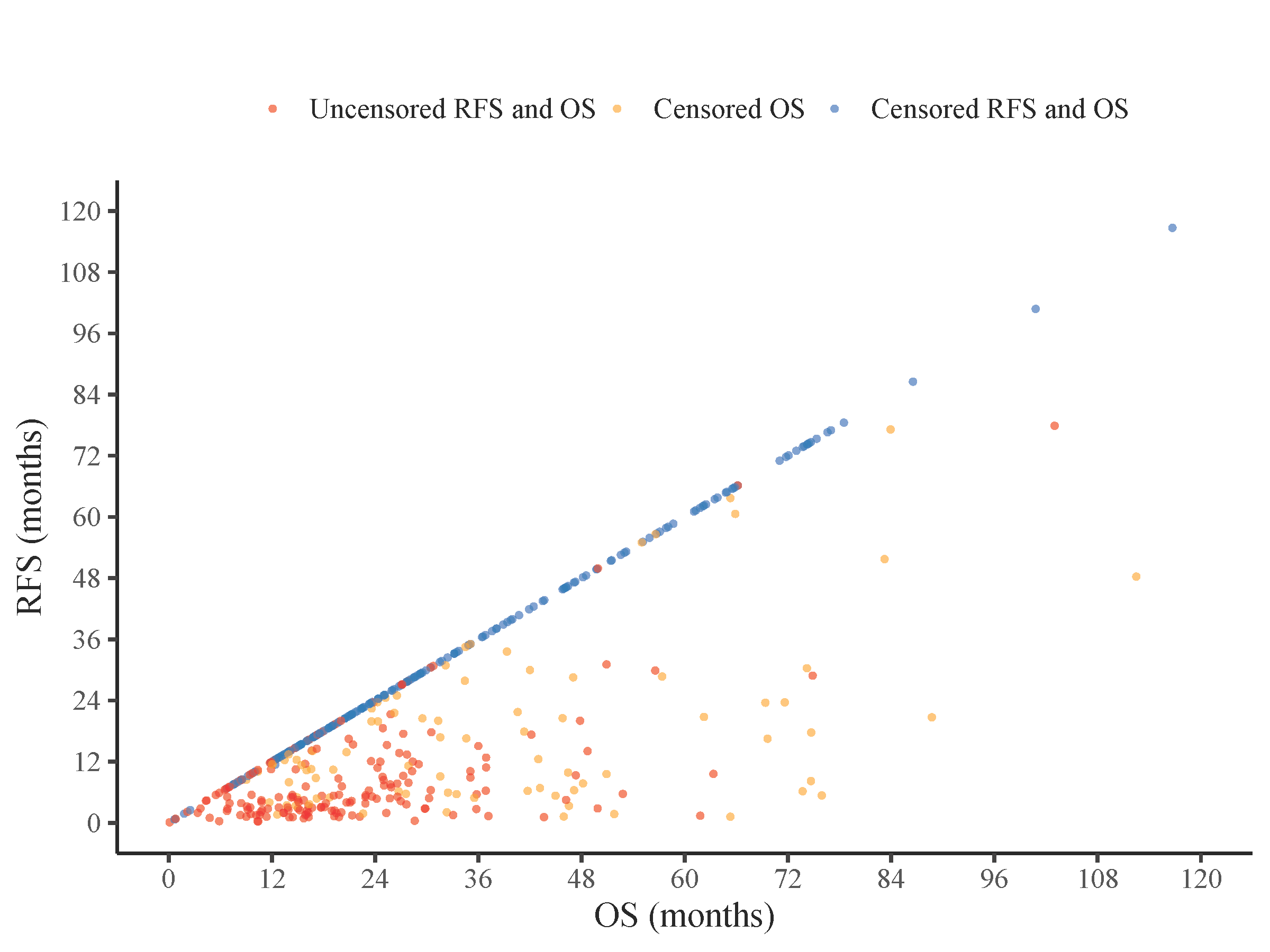

Supplement: Supplementary file 5 — Supplementary file5 (TIF 347 KB) [file 10434_2025_17156_MOESM5_ESM.tif]
