# Supplementary material for: Recurrence-Free Survival as a Surrogate for Overall Survival Among Patients with Intrahepatic Cholangiocarcinoma Following Upfront Surgery: An International Multi-institutional Analysis
Source: Ann Surg Oncol. 2025 Mar 21;32(7):4967–75. doi: 10.1245/s10434-025-17156-5 (PMC12130119; doi:10.1245/s10434-025-17156-5)
Supplement: Supplementary file 6 — Supplementary file6 (DOCX 15 KB) [file 10434_2025_17156_MOESM6_ESM.docx]

Supplementary Figure 1: Kaplan-Meier estimates of survival after recurrence in patients who experienced recurrence.

Supplementary Figure 2: Kaplan-Meier estimates comparing survival after recurrence between patients who experienced recurrence within 36 months and those who had recurrence after 36 months.

Supplementary Figure 3. When assessing the correlation between RFS and OS, a strong correlation was observed in Western countries (ρ = 0.85, 95% CI 0.82–0.87) (Supplementary Figure 3a), whereas a lower correlation was noted in Eastern countries (ρ = 0.65, 95% CI 0.57–0.71) (Supplementary Figure 3b).
